# 1-(2-Hy­droxy­eth­yl)-4-[3-(2-trifluoro­methyl-9*H*-thioxanthen-9-yl­idene)prop­yl]piperazine-1,4-diium dichloride: the dihydro­chloride salt of flupentixol

**DOI:** 10.1107/S1600536811028182

**Published:** 2011-07-23

**Authors:** M. S. Siddegowda, Ray J. Butcher, Mehmet Akkurt, H. S. Yathirajan, B. Narayana

**Affiliations:** a Department of Studies in Chemistry, University of Mysore, Manasagangotri, Mysore 570 006, India; b Department of Chemistry, Howard University, 525 College Street NW, Washington, DC 20059, USA; c Department of Physics, Faculty of Sciences, Erciyes University, 38039 Kayseri, Turkey; d Department of Studies in Chemistry, Mangalore University, Mangalagangotri 574 199, India

## Abstract

In the title compound, C_23_H_27_F_3_N_2_OS^+^·2Cl^−^, the piperazinediium ring adopts a chair conformation. The dihedral angle between the two outer aromatic rings of the 9*H*-thioxanthene unit is 40.35 (18)°. The F atoms in the trifluoro­methyl group are disordered over two sets of sites with occupancies of 0.803 (6) and 0.197 (6). In the crystal, mol­ecules are connected by N—H⋯Cl, O—H⋯Cl C—H⋯O and C—H⋯Cl hydrogen bonds, forming chains propagating along [001]. There are also C—H⋯π inter­actions present in the crystal structure.

## Related literature

For the anti­depressant action of flupentixol, see: Robertson & Trimble (1981 ▶). For related structures, see: Post *et al.* (1975*a* ▶,*b* ▶). For puckering parameters, see: Cremer & Pople (1975 ▶).
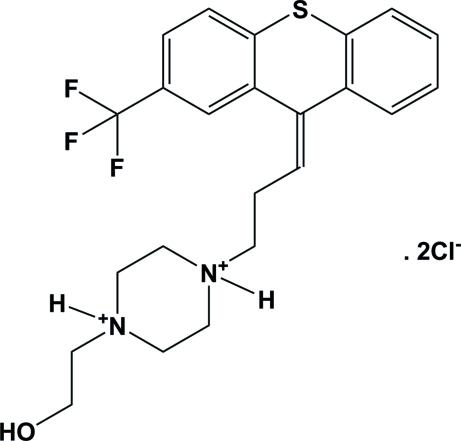


## Experimental

### 

#### Crystal data

C_23_H_27_F_3_N_2_OS^2+^·2Cl^−^
*M* *_r_* = 507.44
Monoclinic, 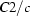

*a* = 34.1750 (17) Å
*b* = 7.1613 (3) Å
*c* = 22.6351 (11) Å
β = 115.307 (6)°
*V* = 5008.0 (5) Å^3^
*Z* = 8
Cu *K*α radiation
μ = 3.46 mm^−1^
*T* = 295 K
0.43 × 0.34 × 0.21 mm

#### Data collection

Oxford Diffraction Xcalibur Ruby Gemini diffractometer
Absorption correction: multi-scan (*CrysAlis PRO*; Oxford Diffraction, 2007 ▶) *T*_min_ = 0.430, *T*_max_ = 1.000
9840 measured reflections
5032 independent reflections
3792 reflections with *I* > 2σ(*I*)
*R*_int_ = 0.028

#### Refinement

*R*[*F* ^2^ > 2σ(*F* ^2^)] = 0.069
*wR*(*F* ^2^) = 0.230
*S* = 1.05
5032 reflections
289 parameters
48 restraints
H-atom parameters constrained
Δρ_max_ = 1.02 e Å^−3^
Δρ_min_ = −0.43 e Å^−3^

### 

Data collection: *CrysAlis PRO* (Oxford Diffraction, 2007 ▶); cell refinement: *CrysAlis PRO*; data reduction: *CrysAlis RED* (Oxford Diffraction, 2007 ▶); program(s) used to solve structure: *SHELXS97* (Sheldrick, 2008 ▶); program(s) used to refine structure: *SHELXL97* (Sheldrick, 2008 ▶); molecular graphics: *SHELXTL* (Sheldrick, 2008 ▶); software used to prepare material for publication: *PLATON* (Spek, 2009 ▶).

## Supplementary Material

Crystal structure: contains datablock(s) global, I. DOI: 10.1107/S1600536811028182/su2290sup1.cif

Structure factors: contains datablock(s) I. DOI: 10.1107/S1600536811028182/su2290Isup2.hkl

Supplementary material file. DOI: 10.1107/S1600536811028182/su2290Isup3.cml

Additional supplementary materials:  crystallographic information; 3D view; checkCIF report

## Figures and Tables

**Table 1:** Hydrogen-bond geometry (Å, °) *Cg*1 and *Cg*2 are the centroids of the C2–C7 and C8–C13 benzene rings, respectively.

*D*—H⋯*A*	*D*—H	H⋯*A*	*D*⋯*A*	*D*—H⋯*A*
N1—H1*A*⋯Cl1^i^	0.91	2.10	2.997 (3)	168
O1—H1*B*⋯Cl1	0.82	2.27	3.030 (4)	155
N2—H2*A*⋯Cl2	0.91	2.13	3.035 (3)	175
C17—H17*A*⋯Cl1^ii^	0.97	2.64	3.600 (4)	169
C18—H18*A*⋯Cl1^iii^	0.97	2.64	3.561 (3)	159
C20—H20*B*⋯O1	0.97	2.34	2.999 (6)	125
C21—H21*A*⋯O1^ii^	0.97	2.35	3.153 (6)	140
C22—H22*A*⋯Cl2^iv^	0.97	2.77	3.690 (4)	160
C19—H19*A*⋯*Cg*2^v^	0.97	2.65	3.618 (4)	176
C23—H23*B*⋯*Cg*1^i^	0.97	2.69	3.658 (5)	174

Symmetry codes: (i) 
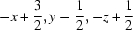
; (ii) 
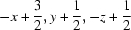
; (iii) 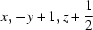; (iv) 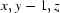; (v) 
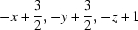
.

**Fig. 1.**
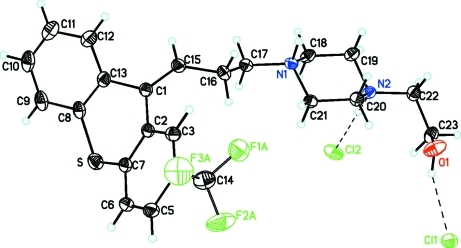
ORTEP view of the title molecule, showing the atom labeling scheme and the displacement ellipsoids drawn at the 30% probability level. Only the major components of the disordered CF3 group are shown.

**Fig. 2.**
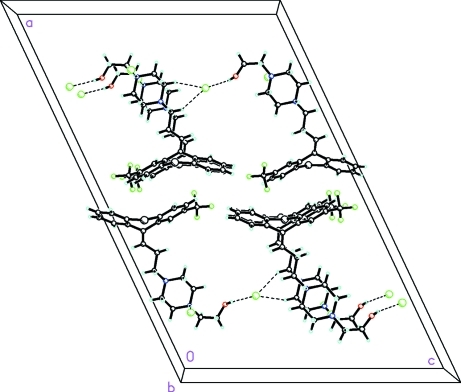
Crystal packing diagram, with the hydrogen bonding (dashed lines), of the title compound viewed along the b axis. Only the major components of the disordered CF3 group are shown.

## supplementary crystallographic information

### Comment

Flupentixol (formally called flupenthixol), 2-(4-(3-(2-(trifluoromethyl)-9H-thioxanthen-9-yl)propyl) piperazin-1-yl)ethanol, is a typical antipsychotic drug of the thioxanthene class. In addition to single drug preparations, it is also available as a deanxit; a combination product containing both melitracen and flupentixol. The antidepressant action of flupentixol has been described by (Robertson & Trimble, 1981). The crystal structures of α-flupenthixol (Post *et al.*, 1975*a*) and β-flupenthixol (Post *et al.*, 1975*b*) have been reported. In view of the importance of flupentixol, herein we report on the crystal structure of its Dihydrochloride salt.

In the molecule of the title compound, (Fig. 1), the piperazinediium ring exhibits a chair conformation, with puckering parameters Q_T_ = 0.584 (4) Å, θ = 5.5 (3) ° and φ = 175 (4) ° (Cremer & Pople, 1975). The two aromatic rings of the 9*H*-thioxanthene unit make a dihedral angle of 40.35 (18)°.

The crystal structure of the title compound is stabilized by N—H···Cl, O—H···Cl, C—H···O and C—H···Cl hydrogen bonds, forming chains propagating along the *c* axis direction (Table 1, Fig. 2). There are also C—H···π interactions present (Table 1).

### Experimental

The title compound was a gift sample from *R. L*. Fine Chemicals, Bangalore, India. X-ray quality crystals were obtained from a 1:1 mixture of ethanol and methanol by slow evaporation (m.p.: 510–512 K).

### Refinement

All the H atoms were placed in calculated positions and treated as riding atoms: N—H(amino) = 0.91 Å, O—H(hydroxyl) = 0.82 Å, C—H = 0.93

and 0.97 Å for aromatic and methylene H-atoms, respectively, with *U*_iso_(H) = k × *U*_eq_(O,C,N), where k = 1.5 for OH(hydroxyl) and k = 1.2 for all other H-atoms. Atoms F1, F2 and F3 of the CF_3_ group are disordered over two sets of sites, with refined occupancy factors in the ratio 0.803 (6):0.197 (6). 14 reflections with bad agreement between F_o_ and F_c_ were omitted from the last cycles of least-squares refinement.

### Crystal data

**Table:**

C_23_H_27_F_3_N_2_OS^2+^·2Cl^−^	*F*(000) = 2112
*M**_r_* = 507.44	*D*_x_ = 1.346 Mg m^−^^3^
Monoclinic, *C*2/*c*	Cu *K*α radiation, λ = 1.54184 Å
Hall symbol: -C 2yc	Cell parameters from 3191 reflections
*a* = 34.1750 (17) Å	θ = 5.2–75.3°
*b* = 7.1613 (3) Å	µ = 3.46 mm^−^^1^
*c* = 22.6351 (11) Å	*T* = 295 K
β = 115.307 (6)°	Prism, colourless
*V* = 5008.0 (5) Å^3^	0.43 × 0.34 × 0.21 mm
*Z* = 8	

### Data collection

**Table:**

Oxford Diffraction Xcalibur Ruby Gemini diffractometer	5032 independent reflections
Radiation source: Enhance (Cu) X-ray Source	3792 reflections with *I* > 2σ(*I*)
graphite	*R*_int_ = 0.028
Detector resolution: 10.5081 pixels mm^-1^	θ_max_ = 75.4°, θ_min_ = 5.5°
ω scans	*h* = −42→41
Absorption correction: multi-scan (*CrysAlis PRO*; Oxford Diffraction, 2007)	*k* = −8→8
*T*_min_ = 0.430, *T*_max_ = 1.000	*l* = −28→17
9840 measured reflections	

### Refinement

**Table:**

Refinement on *F*^2^	Primary atom site location: structure-invariant direct methods
Least-squares matrix: full	Secondary atom site location: difference Fourier map
*R*[*F*^2^ > 2σ(*F*^2^)] = 0.069	Hydrogen site location: inferred from neighbouring sites
*wR*(*F*^2^) = 0.230	H-atom parameters constrained
*S* = 1.05	*w* = 1/[σ^2^(*F*_o_^2^) + (0.1341*P*)^2^ + 2.8648*P*] where *P* = (*F*_o_^2^ + 2*F*_c_^2^)/3
5032 reflections	(Δ/σ)_max_ < 0.001
289 parameters	Δρ_max_ = 1.02 e Å^−^^3^
48 restraints	Δρ_min_ = −0.43 e Å^−^^3^

### Special details

**Table:**

Geometry. Bond distances, angles *etc*. have been calculated using the rounded fractional coordinates. All su's are estimated from the variances of the (full) variance-covariance matrix. The cell e.s.d.'s are taken into account in the estimation of distances, angles and torsion angles
Refinement. Refinement on *F*^2^ for ALL reflections except those flagged by the user for potential systematic errors. Weighted *R*-factors *wR* and all goodnesses of fit *S* are based on *F*^2^, conventional *R*-factors *R* are based on *F*, with *F* set to zero for negative *F*^2^. The observed criterion of *F*^2^ > σ(*F*^2^) is used only for calculating -*R*-factor-obs *etc*. and is not relevant to the choice of reflections for refinement. *R*-factors based on *F*^2^ are statistically about twice as large as those based on *F*, and *R*-factors based on ALL data will be even larger.

### Fractional atomic coordinates and isotropic or equivalent isotropic displacement parameters (Å^2^)

**Table:**

	*x*	*y*	*z*	*U*_iso_*/*U*_eq_	Occ. (<1)
S	0.57461 (4)	1.14222 (16)	0.35221 (7)	0.0884 (4)	
F1B	0.57123 (16)	0.4027 (6)	0.17114 (19)	0.1148 (16)	0.803 (6)
F2B	0.5404 (2)	0.6060 (7)	0.0960 (2)	0.153 (2)	0.803 (6)
F3B	0.50494 (14)	0.4598 (8)	0.1387 (3)	0.156 (2)	0.803 (6)
O1	0.81039 (14)	0.4005 (6)	0.22005 (15)	0.1096 (15)	
N1	0.74387 (7)	0.4815 (4)	0.39823 (12)	0.0526 (7)	
N2	0.81206 (8)	0.4717 (4)	0.35317 (11)	0.0554 (8)	
C1	0.60586 (9)	0.7385 (5)	0.38628 (16)	0.0576 (9)	
C2	0.58158 (6)	0.7828 (3)	0.31479 (8)	0.0575 (9)	
C3	0.57390 (7)	0.6452 (2)	0.26781 (10)	0.0638 (10)	
C4	0.55139 (7)	0.6884 (3)	0.20190 (9)	0.0691 (11)	
C5	0.53658 (8)	0.8693 (4)	0.18297 (8)	0.0803 (14)	
C6	0.54426 (8)	1.0069 (3)	0.22994 (12)	0.0813 (16)	
C7	0.56677 (7)	0.9636 (3)	0.29585 (11)	0.0675 (11)	
C8	0.57315 (10)	1.0114 (6)	0.41747 (19)	0.0704 (11)	
C9	0.55696 (12)	1.0993 (8)	0.4577 (2)	0.0892 (18)	
C10	0.55546 (13)	1.0009 (10)	0.5087 (2)	0.098 (2)	
C11	0.56923 (16)	0.8215 (10)	0.5202 (2)	0.098 (2)	
C12	0.58549 (13)	0.7320 (7)	0.48050 (19)	0.0804 (14)	
C13	0.58772 (10)	0.8272 (6)	0.42855 (17)	0.0639 (10)	
C14	0.54294 (13)	0.5409 (7)	0.15137 (18)	0.0959 (18)	
C15	0.64145 (11)	0.6333 (5)	0.41210 (17)	0.0631 (10)	
C16	0.66663 (10)	0.5476 (5)	0.37845 (17)	0.0623 (10)	
C17	0.71416 (10)	0.5881 (5)	0.41901 (15)	0.0574 (9)	
C18	0.78999 (10)	0.5299 (6)	0.44121 (14)	0.0654 (12)	
C19	0.82119 (10)	0.4273 (6)	0.42243 (15)	0.0669 (12)	
C20	0.76719 (10)	0.4095 (5)	0.31076 (15)	0.0609 (10)	
C21	0.73502 (9)	0.5109 (5)	0.32828 (14)	0.0585 (9)	
C22	0.84554 (11)	0.3868 (6)	0.33492 (18)	0.0703 (13)	
C23	0.84521 (12)	0.4698 (7)	0.27293 (19)	0.0754 (14)	
F3A	0.5493 (6)	0.3669 (14)	0.1704 (10)	0.156 (2)	0.197 (6)
F1A	0.5709 (4)	0.581 (2)	0.1264 (8)	0.1148 (16)	0.197 (6)
F2A	0.5040 (3)	0.556 (3)	0.1030 (6)	0.153 (2)	0.197 (6)
Cl1	0.78205 (5)	0.58962 (13)	0.08841 (5)	0.0878 (4)	
Cl2	0.81966 (4)	0.89329 (16)	0.34901 (7)	0.0992 (4)	
H1B	0.80170	0.47920	0.19100	0.1640*	
H5A	0.52150	0.89820	0.13890	0.0960*	
H2A	0.81320	0.59790	0.34950	0.0670*	
H3A	0.58380	0.52420	0.28050	0.0770*	
H1A	0.74020	0.35790	0.40370	0.0630*	
H11A	0.56790	0.75720	0.55500	0.1180*	
H12A	0.59480	0.60870	0.48890	0.0960*	
H15A	0.65200	0.61010	0.45670	0.0760*	
H16A	0.65730	0.60030	0.33510	0.0750*	
H6A	0.53430	1.12790	0.21730	0.0980*	
H9A	0.54740	1.22240	0.45000	0.1070*	
H10A	0.54480	1.05820	0.53570	0.1170*	
H18A	0.79620	0.50020	0.48620	0.0790*	
H18B	0.79410	0.66320	0.43860	0.0790*	
H19A	0.85060	0.46350	0.45110	0.0800*	
H19B	0.81840	0.29390	0.42710	0.0800*	
H20A	0.76480	0.27620	0.31590	0.0730*	
H20B	0.76080	0.43360	0.26540	0.0730*	
H21A	0.73620	0.64340	0.32030	0.0700*	
H21B	0.70610	0.46690	0.30050	0.0700*	
H22A	0.84050	0.25350	0.32890	0.0840*	
H22B	0.87390	0.40520	0.37060	0.0840*	
H23A	0.84330	0.60480	0.27400	0.0900*	
H23B	0.87180	0.43780	0.26970	0.0900*	
H16B	0.66180	0.41380	0.37420	0.0750*	
H17A	0.71910	0.72070	0.41650	0.0690*	
H17B	0.72140	0.55860	0.46430	0.0690*	

### Atomic displacement parameters (Å^2^)

**Table:**

	*U*^11^	*U*^22^	*U*^33^	*U*^12^	*U*^13^	*U*^23^
S	0.0929 (7)	0.0654 (6)	0.1139 (8)	0.0122 (5)	0.0508 (6)	0.0065 (5)
F1B	0.132 (3)	0.112 (3)	0.093 (2)	0.009 (2)	0.041 (2)	−0.020 (2)
F2B	0.246 (6)	0.139 (3)	0.070 (2)	−0.015 (4)	0.064 (3)	0.007 (2)
F3B	0.129 (3)	0.150 (4)	0.156 (4)	−0.058 (3)	0.030 (3)	−0.033 (3)
O1	0.124 (3)	0.129 (3)	0.0652 (16)	−0.048 (2)	0.0302 (17)	−0.0017 (17)
N1	0.0480 (12)	0.0530 (13)	0.0522 (13)	0.0016 (10)	0.0169 (10)	0.0101 (10)
N2	0.0478 (12)	0.0677 (15)	0.0464 (12)	−0.0014 (11)	0.0159 (9)	0.0046 (11)
C1	0.0474 (14)	0.0607 (17)	0.0644 (16)	−0.0007 (13)	0.0237 (12)	0.0043 (14)
C2	0.0438 (13)	0.0623 (17)	0.0693 (18)	0.0024 (12)	0.0269 (12)	0.0100 (15)
C3	0.0514 (15)	0.0686 (19)	0.0668 (18)	−0.0023 (14)	0.0208 (13)	0.0109 (16)
C4	0.0506 (16)	0.085 (2)	0.0688 (19)	−0.0043 (16)	0.0227 (14)	0.0097 (18)
C5	0.0608 (19)	0.106 (3)	0.075 (2)	0.014 (2)	0.0298 (17)	0.032 (2)
C6	0.077 (2)	0.085 (3)	0.094 (3)	0.027 (2)	0.048 (2)	0.037 (2)
C7	0.0565 (17)	0.072 (2)	0.083 (2)	0.0091 (15)	0.0385 (16)	0.0152 (18)
C8	0.0448 (14)	0.084 (2)	0.079 (2)	0.0041 (15)	0.0231 (14)	−0.0116 (19)
C9	0.0517 (18)	0.114 (4)	0.091 (3)	0.011 (2)	0.0202 (17)	−0.025 (3)
C10	0.0555 (19)	0.153 (5)	0.083 (3)	0.001 (3)	0.0279 (18)	−0.033 (3)
C11	0.078 (3)	0.149 (5)	0.073 (2)	−0.024 (3)	0.037 (2)	−0.011 (3)
C12	0.072 (2)	0.102 (3)	0.068 (2)	−0.010 (2)	0.0307 (17)	−0.002 (2)
C13	0.0429 (13)	0.081 (2)	0.0644 (18)	−0.0036 (14)	0.0198 (12)	−0.0025 (16)
C14	0.096 (3)	0.108 (4)	0.067 (2)	−0.014 (3)	0.019 (2)	−0.004 (2)
C15	0.0567 (16)	0.0662 (19)	0.0625 (17)	0.0038 (14)	0.0217 (14)	0.0051 (15)
C16	0.0529 (16)	0.0620 (18)	0.0651 (18)	0.0104 (14)	0.0185 (13)	0.0022 (15)
C17	0.0579 (16)	0.0559 (17)	0.0552 (15)	0.0031 (13)	0.0212 (13)	0.0033 (13)
C18	0.0523 (15)	0.095 (3)	0.0416 (14)	−0.0110 (16)	0.0130 (11)	0.0023 (15)
C19	0.0426 (14)	0.102 (3)	0.0483 (15)	−0.0018 (15)	0.0119 (11)	0.0122 (16)
C20	0.0485 (15)	0.075 (2)	0.0498 (15)	0.0028 (14)	0.0119 (12)	−0.0014 (14)
C21	0.0493 (14)	0.0712 (19)	0.0456 (14)	0.0073 (14)	0.0112 (11)	0.0058 (13)
C22	0.0511 (16)	0.091 (3)	0.0645 (18)	0.0055 (16)	0.0207 (14)	−0.0011 (17)
C23	0.0643 (19)	0.097 (3)	0.072 (2)	−0.0101 (19)	0.0360 (16)	−0.010 (2)
F3A	0.129 (3)	0.150 (4)	0.156 (4)	−0.058 (3)	0.030 (3)	−0.033 (3)
F1A	0.132 (3)	0.112 (3)	0.093 (2)	0.009 (2)	0.041 (2)	−0.020 (2)
F2A	0.246 (6)	0.139 (3)	0.070 (2)	−0.015 (4)	0.064 (3)	0.007 (2)
Cl1	0.1424 (10)	0.0536 (5)	0.0658 (5)	0.0110 (5)	0.0430 (6)	−0.0025 (4)
Cl2	0.0772 (6)	0.0709 (6)	0.1183 (9)	−0.0076 (5)	0.0121 (6)	0.0168 (6)

### Geometric parameters (Å, °)

**Table:**

S—C7	1.745 (3)	C11—C12	1.398 (7)
S—C8	1.768 (4)	C12—C13	1.389 (6)
F1A—C14	1.333 (16)	C15—C16	1.503 (5)
F1B—C14	1.321 (7)	C16—C17	1.513 (5)
F2A—C14	1.318 (13)	C18—C19	1.498 (5)
F2B—C14	1.305 (6)	C20—C21	1.505 (5)
F3A—C14	1.306 (12)	C22—C23	1.520 (6)
F3B—C14	1.337 (7)	C3—H3A	0.9300
O1—C23	1.370 (6)	C5—H5A	0.9300
O1—H1B	0.8200	C6—H6A	0.9300
N1—C18	1.497 (4)	C9—H9A	0.9300
N1—C21	1.494 (4)	C10—H10A	0.9300
N1—C17	1.498 (5)	C11—H11A	0.9300
N2—C19	1.496 (4)	C12—H12A	0.9300
N2—C20	1.488 (4)	C15—H15A	0.9300
N2—C22	1.501 (5)	C16—H16A	0.9700
N1—H1A	0.9100	C16—H16B	0.9700
N2—H2A	0.9100	C17—H17A	0.9700
C1—C13	1.487 (5)	C17—H17B	0.9700
C1—C2	1.503 (4)	C18—H18A	0.9700
C1—C15	1.335 (5)	C18—H18B	0.9700
C2—C7	1.390 (3)	C19—H19A	0.9700
C2—C3	1.390 (3)	C19—H19B	0.9700
C3—C4	1.390 (3)	C20—H20A	0.9700
C4—C14	1.492 (5)	C20—H20B	0.9700
C4—C5	1.390 (4)	C21—H21A	0.9700
C5—C6	1.390 (3)	C21—H21B	0.9700
C6—C7	1.390 (3)	C22—H22A	0.9700
C8—C13	1.394 (6)	C22—H22B	0.9700
C8—C9	1.401 (6)	C23—H23A	0.9700
C9—C10	1.372 (7)	C23—H23B	0.9700
C10—C11	1.355 (10)		
			
C7—S—C8	99.91 (17)	N2—C22—C23	113.0 (3)
C23—O1—H1B	109.00	O1—C23—C22	109.0 (4)
C17—N1—C21	113.8 (2)	C2—C3—H3A	120.00
C18—N1—C21	109.6 (2)	C4—C3—H3A	120.00
C17—N1—C18	110.2 (3)	C4—C5—H5A	120.00
C19—N2—C22	111.1 (3)	C6—C5—H5A	120.00
C20—N2—C22	113.4 (3)	C5—C6—H6A	120.00
C19—N2—C20	108.0 (3)	C7—C6—H6A	120.00
C17—N1—H1A	108.00	C8—C9—H9A	121.00
C18—N1—H1A	108.00	C10—C9—H9A	121.00
C21—N1—H1A	108.00	C9—C10—H10A	119.00
C19—N2—H2A	108.00	C11—C10—H10A	120.00
C20—N2—H2A	108.00	C10—C11—H11A	120.00
C22—N2—H2A	108.00	C12—C11—H11A	120.00
C13—C1—C15	120.8 (3)	C11—C12—H12A	120.00
C2—C1—C13	114.4 (3)	C13—C12—H12A	120.00
C2—C1—C15	124.8 (3)	C1—C15—H15A	116.00
C3—C2—C7	119.99 (17)	C16—C15—H15A	116.00
C1—C2—C3	120.7 (2)	C15—C16—H16A	110.00
C1—C2—C7	119.3 (2)	C15—C16—H16B	110.00
C2—C3—C4	120.01 (16)	C17—C16—H16A	110.00
C3—C4—C5	119.98 (17)	C17—C16—H16B	110.00
C3—C4—C14	120.2 (2)	H16A—C16—H16B	108.00
C5—C4—C14	119.9 (2)	N1—C17—H17A	109.00
C4—C5—C6	120.01 (17)	N1—C17—H17B	109.00
C5—C6—C7	120.0 (2)	C16—C17—H17A	109.00
S—C7—C2	122.40 (17)	C16—C17—H17B	109.00
S—C7—C6	117.57 (17)	H17A—C17—H17B	108.00
C2—C7—C6	120.0 (2)	N1—C18—H18A	109.00
S—C8—C13	121.1 (3)	N1—C18—H18B	109.00
C9—C8—C13	121.2 (4)	C19—C18—H18A	109.00
S—C8—C9	117.7 (3)	C19—C18—H18B	109.00
C8—C9—C10	118.9 (5)	H18A—C18—H18B	108.00
C9—C10—C11	120.9 (5)	N2—C19—H19A	110.00
C10—C11—C12	120.8 (5)	N2—C19—H19B	110.00
C11—C12—C13	120.0 (5)	C18—C19—H19A	110.00
C1—C13—C12	121.6 (4)	C18—C19—H19B	110.00
C1—C13—C8	120.2 (3)	H19A—C19—H19B	108.00
C8—C13—C12	118.2 (4)	N2—C20—H20A	110.00
F1B—C14—F2B	109.4 (5)	N2—C20—H20B	109.00
F1B—C14—F3B	104.5 (5)	C21—C20—H20A	110.00
F1B—C14—C4	113.4 (3)	C21—C20—H20B	110.00
F2B—C14—F3B	106.8 (5)	H20A—C20—H20B	108.00
F2B—C14—C4	113.3 (4)	N1—C21—H21A	109.00
F3B—C14—C4	108.9 (4)	N1—C21—H21B	109.00
F3A—C14—C4	118.2 (9)	C20—C21—H21A	109.00
F1A—C14—C4	103.5 (7)	C20—C21—H21B	109.00
F2A—C14—C4	112.0 (9)	H21A—C21—H21B	108.00
F2A—C14—F3A	108.6 (13)	N2—C22—H22A	109.00
F1A—C14—F2A	106.4 (9)	N2—C22—H22B	109.00
F1A—C14—F3A	107.3 (12)	C23—C22—H22A	109.00
C1—C15—C16	128.4 (3)	C23—C22—H22B	109.00
C15—C16—C17	108.2 (3)	H22A—C22—H22B	108.00
N1—C17—C16	114.1 (3)	O1—C23—H23A	110.00
N1—C18—C19	112.4 (3)	O1—C23—H23B	110.00
N2—C19—C18	109.8 (3)	C22—C23—H23A	110.00
N2—C20—C21	110.7 (3)	C22—C23—H23B	110.00
N1—C21—C20	111.8 (3)	H23A—C23—H23B	108.00
			
C8—S—C7—C2	−31.8 (3)	C3—C2—C7—C6	0.0 (4)
C8—S—C7—C6	147.3 (2)	C2—C3—C4—C5	0.0 (4)
C7—S—C8—C9	−148.7 (3)	C2—C3—C4—C14	−179.8 (3)
C7—S—C8—C13	31.5 (4)	C3—C4—C5—C6	0.0 (4)
C18—N1—C17—C16	−179.7 (3)	C14—C4—C5—C6	179.8 (3)
C21—N1—C17—C16	56.7 (4)	C3—C4—C14—F1B	−25.0 (5)
C17—N1—C18—C19	−179.9 (3)	C3—C4—C14—F2B	−150.5 (4)
C21—N1—C18—C19	−53.9 (4)	C3—C4—C14—F3B	90.8 (4)
C17—N1—C21—C20	176.9 (3)	C5—C4—C14—F1B	155.2 (4)
C18—N1—C21—C20	53.0 (4)	C5—C4—C14—F2B	29.8 (6)
C20—N2—C19—C18	−60.8 (4)	C5—C4—C14—F3B	−89.0 (4)
C22—N2—C19—C18	174.2 (3)	C4—C5—C6—C7	0.0 (4)
C19—N2—C20—C21	60.8 (4)	C5—C6—C7—S	−179.1 (2)
C22—N2—C20—C21	−175.6 (3)	C5—C6—C7—C2	0.0 (4)
C19—N2—C22—C23	−164.1 (3)	S—C8—C9—C10	180.0 (4)
C20—N2—C22—C23	74.0 (4)	C13—C8—C9—C10	−0.2 (6)
C13—C1—C2—C3	−138.9 (3)	S—C8—C13—C1	1.5 (5)
C13—C1—C2—C7	41.1 (4)	S—C8—C13—C12	−179.7 (3)
C15—C1—C2—C3	42.8 (5)	C9—C8—C13—C1	−178.4 (4)
C15—C1—C2—C7	−137.2 (3)	C9—C8—C13—C12	0.5 (6)
C2—C1—C13—C8	−41.3 (5)	C8—C9—C10—C11	−0.2 (7)
C2—C1—C13—C12	139.9 (4)	C9—C10—C11—C12	0.2 (8)
C15—C1—C13—C8	137.1 (4)	C10—C11—C12—C13	0.1 (7)
C15—C1—C13—C12	−41.7 (6)	C11—C12—C13—C1	178.4 (4)
C2—C1—C15—C16	4.3 (6)	C11—C12—C13—C8	−0.4 (6)
C13—C1—C15—C16	−173.9 (4)	C1—C15—C16—C17	132.6 (4)
C1—C2—C3—C4	180.0 (2)	C15—C16—C17—N1	169.2 (3)
C7—C2—C3—C4	0.0 (4)	N1—C18—C19—N2	58.8 (4)
C1—C2—C7—S	−0.9 (3)	N2—C20—C21—N1	−58.1 (4)
C1—C2—C7—C6	−180.0 (3)	N2—C22—C23—O1	−75.4 (5)
C3—C2—C7—S	179.1 (2)		

### Hydrogen-bond geometry (Å, °)

**Table:**

Cg1 and Cg2 are the centroids of the C2–C7 and C8–C13 benzene rings, respectively.

**Table:**

*D*—H···*A*	*D*—H	H···*A*	*D*···*A*	*D*—H···*A*
N1—H1A···Cl1^i^	0.91	2.10	2.997 (3)	168
O1—H1B···Cl1	0.82	2.27	3.030 (4)	155
N2—H2A···Cl2	0.91	2.13	3.035 (3)	175
C17—H17A···Cl1^ii^	0.97	2.64	3.600 (4)	169
C18—H18A···Cl1^iii^	0.97	2.64	3.561 (3)	159
C20—H20B···O1	0.97	2.34	2.999 (6)	125
C21—H21A···O1^ii^	0.97	2.35	3.153 (6)	140
C22—H22A···Cl2^iv^	0.97	2.77	3.690 (4)	160
C19—H19A···Cg2^v^	0.97	2.65	3.618 (4)	176
C23—H23B···Cg1^i^	0.97	2.69	3.658 (5)	174

Symmetry codes: (i) −*x*+3/2, *y*−1/2, −*z*+1/2; (ii) −*x*+3/2, *y*+1/2, −*z*+1/2; (iii) *x*, −*y*+1, *z*+1/2; (iv) *x*, *y*−1, *z*; (v) −*x*+3/2, −*y*+3/2, −*z*+1.
